# Solid-pseudopapillary Neoplasms of the Pancreas is still an Enigma: a Clinicopathological Review

**DOI:** 10.1007/s12253-019-00671-8

**Published:** 2019-06-17

**Authors:** Attila Zalatnai, Viktória Kis-Orha

**Affiliations:** First Department of Pathology and Experimental Cancer Research, Faculty of Medicine, Semmelweis University, H-1085 Üllői út, Budapest, 26 Hungary

**Keywords:** Pancreas, Solid-pseudopapillary neoplasm, Histogenesis, Review

## Abstract

The solid-pseudopapillary neoplasm of the pancreas is a rare but enigmatic entity occurring mainly in young women. Since the first description by V. Frantz in 1959 the terminology of this tumor has continuously changed but it has remained simply descriptive, because the exact histogenesis is still obscure. Although in majority of cases the survival is excellent, nevertheless, the expected prognosis is not exactly predictable. In this review the authors aim to summarize its clinico-pathological features, the expected biological behavior, the molecular alterations, the immune phenotype and discuss the putative histogenesis. From diagnostic point of view, the salient histological characteristic findings are analyzed that would help to differentiate it from other, look-alike pancreatic tumors, and suggestions are made about the desirable content of the histological report.

## Introduction

Since the first description of a peculiar pancreatic tumor in 1959 [1], its terminology has been changed many times indicating that the exact nature and origin of this entity has remained speculative. In the original description the tumor was classified as a benign exocrine glandular lesion, most probably a papillary cystadenoma, although it was stated that ″accurate interpretation of these neoplasms is difficult”. It has long been disregarded since the 1978 WHO did not even mention. In the former literature it could be found under various names (solid-cystic tumor, solid-cystic acinar tumor, papillary-cystic tumor, solid-papillary tumor), but indeed, the actually used” *solid- pseudopapillary neoplasm*” (SPN) is similarly merely a descriptive designation denoting the morphological features, but leaving the histogenesis open.

## Clinical Findings

The SPN is a rare pancreatic tumor: during 10 to 35 years of period various institutes have reported 9–187 pathologically diagnosed cases [2–5]. It may occur in both sexes, with a female predominance. Familiarity is not a feature. The median age is about 25–35 years, but children and old patients are equally affected; it may occur in an 8-year-old child up to 75 years of age. (Between 2007 and 2018 at our institute 13 SPNs have been diagnosed, all of them were female (11–59 years). The clinical signs are typically insignificant: it may be painless, or the patients may just complain of an undefined, slight, upper abdominal pain. It is not accompanied by general symptoms, hormonal activity is absent, the lab findings are normal; therefore, early diagnosis is usually not possible. An exceptional childhood case (a ruptured SPN following a blunt abdominal trauma) was reported by *Tajima* et al., but later on this tumor proved to be malignant [9]. A spontaneous rupture in a pregnant woman was also documented [10], but there is no evidence that the pregnancy itself could facilitate breakup in SPN. Regarding the localization, there is no preference of it; any part of the pancreas can be the site of origin. Although it is a primary pancreatic neoplasm, exceedingly rarely it may also occur in extrapancreatic places (omentum, adrenal or mesentery) [11–13]. As a rule, the neoplasm presents as a solitary lesion, the multicentric manifestation is a curiosity [14].

Although SPN does occur in females and males, some gender differences are observed. The affected males are usually older, but the tumor size, the location or the clinical symptoms do not differ significantly [3, 15].

## Pathological Characteristics

**Fig. 1:**
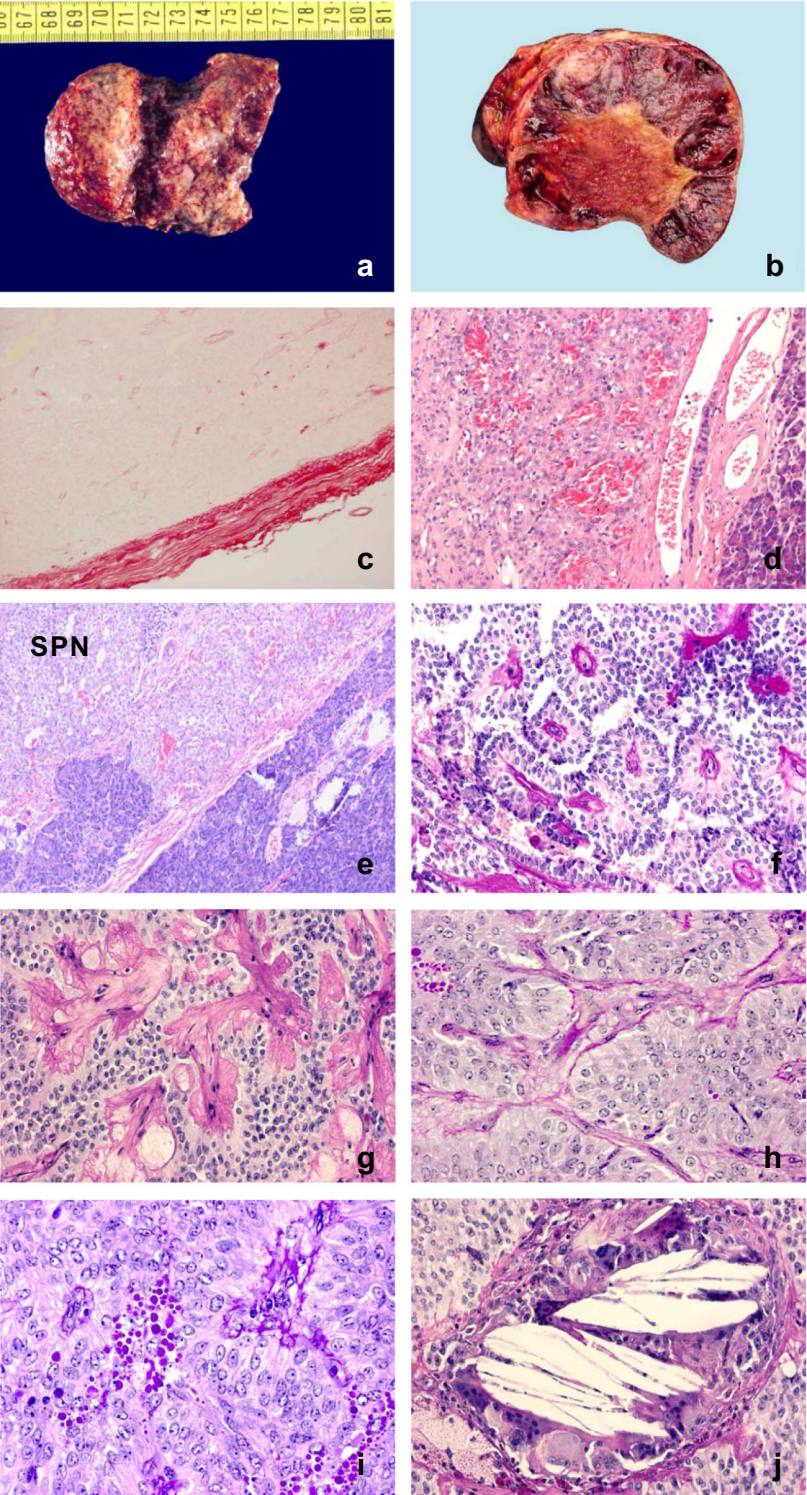
Pathological characteristics of solid-pseudopapillary neoplasm. **a** The tumor is sharply demarcated; **b** Circumscribed tumor with necrotic-hemorrhagic, degenerative changes; **c** Connective tissue capsule around the tumor (Picrosirius red, ×100); **d** Rich vascularization is seen (HE, ×100); **e** Solid pattern (HE, ×100); **f** pseudopapillary pattern (PAS, ×200); **g** Hyalinized stroma (PAS, ×200); **h** Bland, monomorphous nuclei (PAS, ×200); **i** Numerous, hyalinic globules (PAS, ×200); **j** Cholesterol crystals with multinucleated giant cells (PAS, ×100)

Because the clinical symptoms are usually vague or even the tumor can be an incidental finding, at the time of discovery it may be bulky; the mean size is about 6–8 cm, and the diameter may reach up to 15–22 cm [6–8]. Macroscopically, the sharp demarcation from the pancreatic tissue is typical, making the surgical removal easy (Fig. 1a) Sometimes it can be surrounded by a delicate or thick capsule. By palpation it has a rubbery feeling, and the cut surface is rather characteristically spongy in appearance. When the tumor is voluminous, extensive necrotic and hemorrhagic areas may be seen. (Fig. 1b).

**Fig. 2:**
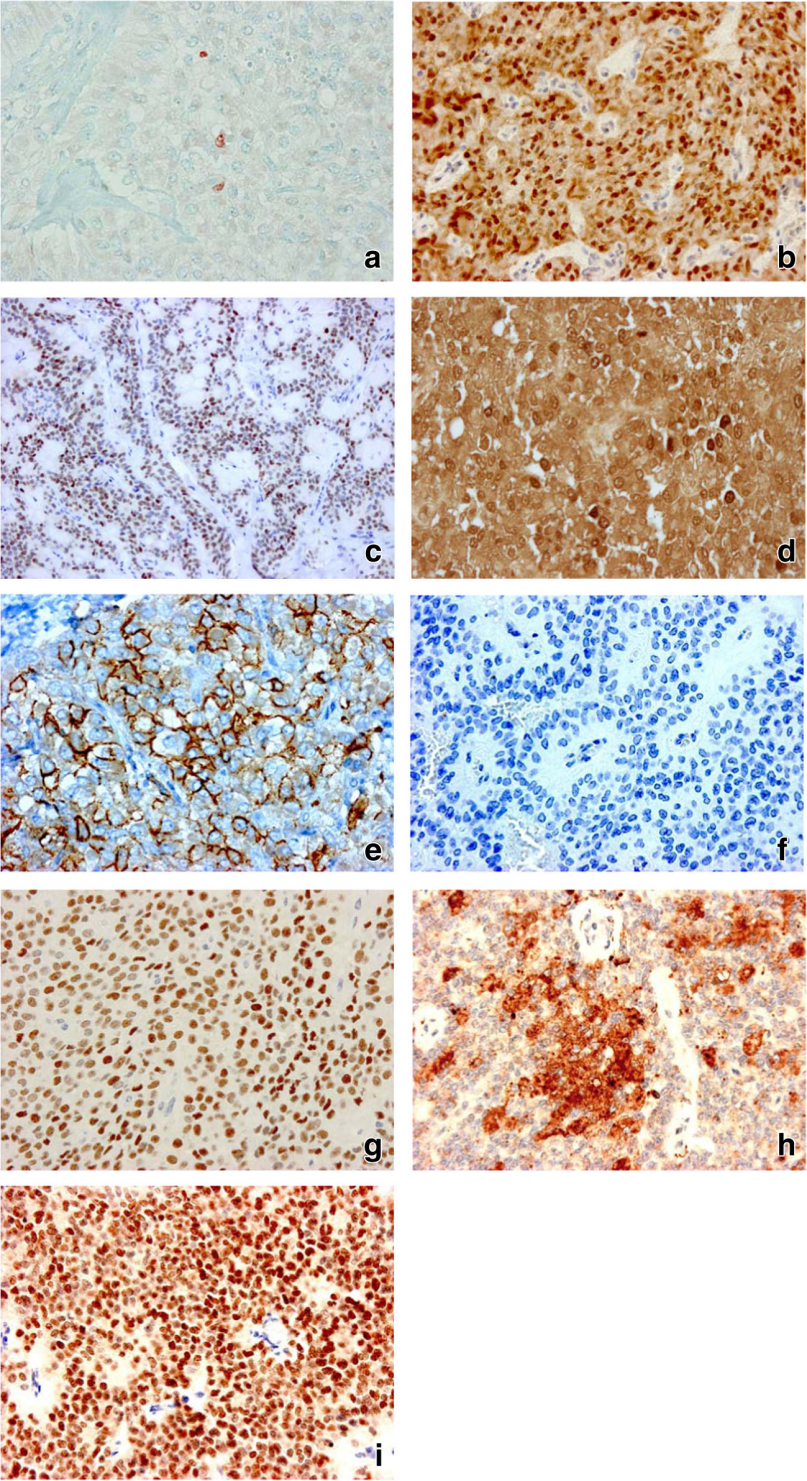
Immunohistochemical characteristics of solid-pseudopapillary neoplasm. **a** very low Ki-67 score (×200); **b** nuclear β-catenin expression (×100); **c** cyclin D1 expression (×100); **d** AAT positivity (×200); **e** CD56 expression (×400); **f** loss of E-cadherin (×200); **g** Progesteron receptor positive expression (×100); **h** CD99 expression (×200); **i** SOX-11 nuclear positivity (×100)

Histologically, according to the terminology, two tissue patterns are observed: large areas of solid sheets of cells are randomly mixed with pseuodopapillary structures (Figs. 1e-f). The separation from the pancreatic tissue is sharp, or it displays a collagenous capsule (Fig. 1c). Plenty of vascular channels are seen, and in the stroma variable amount of hyalinized areas are noted (Figs.1d, g). The cells are pale, roundish, characteristically monomorphous, the nuclei are oval in shape and are frequently grooved, the nucleoli are marginated (Fig. 1h). In some areas the cells may have a foamy cytoplasm. A frequent, rather typical feature is the presence of PAS-positive globules in grouping, and the cholesterol clefts are also commonly seen (Figs. 1i-j). Mitotic figures are rarely seen (0–6/20 HPF) with no occurrence of atypical forms, and the Ki-67 score is very low (Fig. 2a). Presence of psammoma bodies is a rare finding.

A conspicuous but unexplained phenomenon was reported by Japanese authors comparing SPN in females and males. They could not observe fibrous capsule and cholesterol clefts in the male tumors, but these histological signs were seen in more than 60% among females. There were slight, but not significant differences regarding the capillary density, but the cystic degenerations or the necrotic areas occurred at the same frequency [15].

Very rarely, peculiar, pigmented variants may also occur [16, 17]. In these cases either a large amount of lipofuscin or melanin is accumulated.

## Molecular Characteristics

Several studies are available about the molecular alterations in SPN [18–23]. The tumor has complex karyotypic changes involving chromosomes 2, 4 or X, including breakpoints, bands or monosomy. Loss of heterozygosity for HRAS was also identified [18]. Hundreds to thousands of genes are differently expressed among them tumor associated genes. Most papers underline the importance of disrupted Wnt/β-catenin signaling pathways with concomitant cyclin D1 overexpression. Characteristic finding is the mutation in exon 3 of CTNNB1, but activated Hedgehog, androgen receptor, epithelial-mesenchymal transition (EMT)-coupled genes have also been identified, and several, closely associated miRNAs, especially the miR-200 family. Upregulated p27, p21 are typical, but no p53 or K-ras mutations are present. The ErbB and GnRH signaling pathways are also disturbed.

Proteomic profiles were also examined with high-resolution mass spectrometry [24]. More than 300 differentially expressed (both up- and downregulated) proteins have been identified. In addition to the proteins involved in Wnt-signaling like FUS or NONO, overexpressed molecules involved in glycolysis, including HK1, ENO2, PKM2 were found in accordance with their mRNA levels. The presented data indicate that SPN is a distinct pancreatic entity.

## Biological Behavior

In the earlier international classifications SPN was positioned into the group of borderline tumors, but recently the WHO categorizes it as a malignant neoplasm. The scene is, however, intriguing, because the expected biology is unpredictable.

It is generally accepted that the majority of the tumor runs a benign course. In large-scale studies 2.1–22.8% of the treated SPNs proved to be malignant based on local recurrences or metastases [5, 25–28]. The 5-year survival is excellent, reaching 93.6–98.8% [26, 29, 30], and even the 10-year disease-specific survival rate is 96% [31]. The local recurrence rate is low (less than 10% after 5 years of resection) [31]. Women and men have the same prognosis [3] and the multicentric forms have similar clinical and pathologic features to the solitary ones [14]. However, when the tumors are located in the head, the disease-free and the overall survivals are significantly shorter than those of other locations [32]. When metastases develop, they usually occur late, 8 to 15.8 years after complete resection of the primary [21, 31, 33, 34]. This indolent behavior might be due to diploid DNA content and a long (765 days) doubling time [11, 35].

## Solid-Pseudopapillary Carcinoma. Signs of Malignancy

A minority of SPN recurs or even metastasizes despite the bland histological appearance. Although the local recurrence may lead to death, but most of fatal cases arise from liver metastases. Interestingly, the bones, the lungs are not involved, the peritoneal carcinosis is also rarely seen [36, 37]. Widespread metastatic spread (liver, stomach, adrenal gland, lymph node, peritoneum) is even more infrequent event [38].

A burning issue is how to predict the potentially malignant behavior of this tumor, in absence of frankly worrisome histological signs? Unfortunately, no clear-cut answer exists, there are various approaches in the literature, sometimes with alternate results. Some authors suggested that the large tumor size (> 5 cm) and the young age increase the risk of recurrence [28, 39, 40]. It seems logical that the inadequate resection, the positive surgical margins, capsular invasion or even the tumor rupture would be an important predictor [28, 31, 40–42], but some other authors have found that these findings have no predictive value [8, 39]. While the perineural invasion is a classical pathohistological sign in the malignant tumors, this finding is not a reliable indicator of aggressiveness in SPN [8, 39]. Similarly, the mitotic rate or the cellular atypia do not necessarily forecast the recurrence [8].

The tumor typically shows a very low proliferative activity; the Ki-67 rate is 1–2%. Some authors have claimed, when the neoplasm displays an elevated Ki-67 score, a malignant course is expected: high values were detected in 66.7% in malignant cases, but only 8.4% when the tumor was classified as benign (*p* < 0.001) [27]. *Yang* et al. have found that the ≥4% score was associated with poorer recurrence-free survival and in these patients an adverse outcome was expected [29]. In another study this cut-off level was 5% [43].

Some other, potential predictive signs have been published, but still they need further reinforcement. Such a marker could be the galectin-3, which is useful to distinguish SPN from neuroendocrine tumors, but it is also interesting that its low immunohistochemical expression is associated with metastatic spreading [44]. Although the molecular mutation profiles are rarely studied at this context, in a single malignant SPN an uncommon EGFR mutation was identified at L861Q by pyrosequencing [45].

Despite all the above mentioned findings, the assessment of the potentially malignant behavior of SPN remains unpredictable. Even the most careful pathological examination cannot exactly identify patients who may develop recurrence after curative surgical operation. Suggestive signs are capsular infiltration, perineural or vascular invasion, brisk mitotic activity, striking polymorphism, but the absence of these features is not exclusive for malignancy [46, 47].

## Immune Profile

The classical histopathological appearance usually allows the correct diagnosis, but some pancreatic tumors may show similarities and for differential diagnostic reason various complex immunohistochemical examinations are needed. In addition, these markers may serve aids to the possible histogenesis of SPN.

In the literature a large number of antibodies have been checked, but sometimes the results seem to be conflicting. There is a general agreement that practically all of these tumors are positive for vimentin [7. 31, 48, 49], β-catenin [7, 31, 43, 49, 50] (Fig. 2b), cyclin D1 [31, 50] (Fig. 2c), alpha-1-antitrypsin (AAT) [11, 48] (Fig. 2d) or CD56 [12, 17, 31, 51] (Fig. 2e). The loss of E-cadherin expression is also a typical finding [49, 52] (Fig. 2f). Presence of progesterone receptor is regularly detected [8, 12, 31, 48, 50] (Fig. 2g), androgen receptor is expressed in about 80% of cases [43], but the estrogen receptor is usually negative [48, 50, 53]. The cytokeratin expression is highly variable, between 28 to 70% [7, 8, 48], but the CK7 is negative [49]. Most SPNs are positive for CD10, usually with a perinuclear dot pattern [7, 8, 31, 51], and the CD99 was also reported as a reliable positive marker [49, 52] (Fig. 2h). Among the neuroendocrine markers the chromogranin-A is usually absent [7, 48, 50], the NSE (although its specificity is questionable) may either be negative or consistently positive [6, 48], while in a small percentage of tumors synaptophysin is detectable [8, 48]. Because SPN is not regarded as a hormonally active tumor, specific pancreatic hormones are not found in the cells [50]. The TFE3, a transcription factor promoting several genes involved in cell proliferation and growth, proved to be a highly sensitive marker (75–96% of cases), and can be used for reinforcement of the SPN diagnosis [43, 54, 55].

There are some other, seemingly unrelated immune positivities (LEF-1, FUS, WIF-1, CD200) that are expressed with a high frequency [43, 56], but their significance and applicability are far from obvious in this tumor.

The SPN, the neuroendocrine tumors and the acinar cell carcinomas sometimes may show similar histological patterns making their distinction difficult. Based on the immunohistochemical staining properties, some differential diagnostic approaches have been advised. *TFE3* expression, for example, is very frequent (94%) in SPN, while the pancreatic neuroendocrine tumors (PNET) are positive just in 25%, and the acinar carcinomas are negative [55]. Similarly, *β-catenin, glypican-3* or *galectin-3* are positive in SPN, but the neuroendocrine tumors are negative [44, 54, 57]. A new finding was recently reported by *Shen* et al.*,* that the *α-Methylacyl-CoA racemase* (AMACR, P504S), which marker is a very useful diagnostic tool in urological malignancies, is positively stained in SPN, but not in the others [58]. *CD200*, however, is highly expressed either in SPN or in PNET (100% vs. 96%), so it cannot be used for differential diagnosis [56].

## Histogenesis

Despite the extensive investigations, the histogenesis has remained obscure and still hypothetical, because the results from different studies are conflicting. Although vimentin, AAT or NSE are positive in most cases, this phenotype in not specific and does not define a specific lineage [59]. The electron microscopical (EM) studies would be a good tool in this context, however, the findings are incongruent. The presence of zymogen granules and the positive trypsin-stain would indicate an acinar character [6, 60, 61], but other authors failed to detect them [53, 62]. In the vast majority of studies, no neurosecretory granules were found [53, 60, 62], only occasional studies claimed their presence in about 50% of the cells [63]. The strong galectin-3 expression also differentiates it from the galectin-3-negative neuroendocrine tumors [44]. The ductal origin is similarly very unlikely: ultrastructurally the tumors cells do not display ductal cell character [61], K-ras mutation is not detectable [22], the CK19 – which is a characteristically positive marker in conventional adenocarcinoma – is also absent [64]. Based on both immunohistochemical and ultrastructural features, *Kallichandra* et al. proposed that the centroacinar cells would be origin of SPN [65, 66], although the CK7 is negative in this tumor, and among our cases only 1 out of 13 displayed just 10% positivity of the cells. The neural crest [16] or the genital ridge/ovarian anlage attached to the pancreatic tissue [48] have also been proposed. Because of the highly divergent immunohistochemical findings, it fails to reveal a definite phenotypic relationship with any clearly defined lineage [48, 50], some authors suggest that SPN may originate from totipotent primordial cells that would further differentiate toward duct epithelial, acinar or endocrine patterns [55, 66]. This would explain the different immune profiles behind the identical morphology. This hypothesis, however, does not lie on solid evidence, because the genes expressed during pancreatic organogenesis and determine the lineage developmental routes are not regularly identifiable in SPN. So, the histogenesis still remained enigmatic.

## Therapeutic Considerations

Because the SPN mostly appears as a localized tumor, the surgery remains the mainstay of therapy, because the well-circumscribed tumors usually show a favorable prognosis [67]. In the recurrent or metastatic cases, however, various other, but not standardized techniques are available. Among them a tyrosine kinase inhibitor sunitinib and hepatic arterial embolisation [68], hyperthermic intraperitoneal chemotherapy are reported [33, 40], but there are occasional limited experience about the palliative radiotherapy or even liver transplantation [69, 70].

## Important Practical Pathological Considerations

Although in the majority of cases the pathological diagnosis of SPN is quite easy, the major differential diagnostic challenges are the neuroendocrine tumor and the acinar cell carcinoma, because their morphology may sometimes overlap. Although the aspecific NSE is usually positive, but the chromogranin-A or synaptophysin are negative, moreover, no hormonal activity is identified in this tumor. The nuclear expression of β-catenin, SOX-11 and TFE3 positivities are characteristic findings in SPN [71], but not in PNET and in acinar cell carcinoma (Fig. 2i). Similarly, the rarely used claudin 5 and 7 can also distinguish these entities [72]. The loss of E-cadherin immune staining is also a reliable, typical reaction in SPN. In addition, *Geers* et al. have found galectin-3 as a useful positive marker, which is not found in the neuroendocrine tumors [44], similarly to CD99 [49. 52]. The major points are presented in Table 1.

**Table 1:** Differential diagnostic approaches among the look-alike pancreatic lesions

	SPN	PNET	ACC
β-catenin	nuclear, +++	negative	negative
TFE3	94%	25%	negative
E-cadherin	loss of positivity	positive	positive
galectin-3	positive	negative	negative
CD56	positive	positive	negative
claudin 5	positive	negative	negative
claudin 7	±	+++	+++
SOX-11	positive	negative	negative
Pdx-1	negative	positive	positive
CD200	100%	96%	12,5% (focal)

*SPN* solid-pseudopapillary neoplasm, *PNET* pancreatic neuroendocrine tumor, *ACC* acinar cell carcinoma

Another pathological challenge is the assessment of its biological behavior, because despite the bland histology it may run a malignant course, and the classical signs of malignancy are not always present. The age, gender or even multiplicity do not seem to be decisive factors [3, 14, 73], but they should also be taken into account. Although it is not possible to foresee the expected prognosis with absolute certainty, the histological report should contain the (a) size, (b) presence/absence of capsule, (c) free/infiltrated surgical margins, (d) cellular atypia, (e) mitotic activity, (f) perineural/vascular infiltration, (g) expression of galectin-3, and the (h) Ki-67 score.

## Conclusion

The rare solid-pseudopapillary neoplasm may develop in any age and in both sexes, but mainly young women are affected. Although the survival rate is usually high, however, the histological picture does not allow precisely predicting the expected biological behavior. There are some suspicious morphological signs, however, late recurrence, metastases or even death can occur with totally bland appearance. The exact histogenesis remains indeterminate, probably primordial cell underwent divergent differentiation; however, SPN is still an enigmatic tumor warranting further elucidation.
